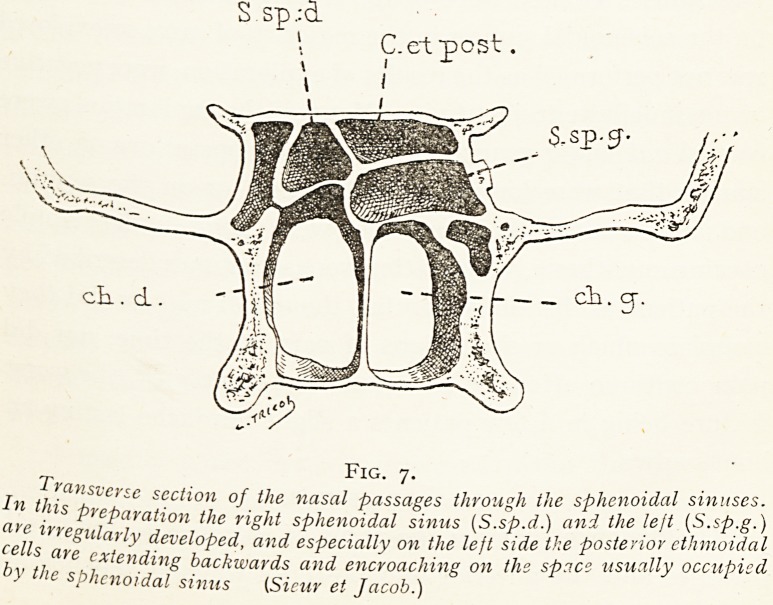# Note on the Technique of Sphenoidal Sinus Exploration for Meningococcal and Other Infections

**Published:** 1916-07

**Authors:** P. Watson-Williams

**Affiliations:** Major, R.A.M.C.(T.); Hon.Consultant for Diseases of Ear, Nose and Throat to the Military Hospitals, Southern Command


### NOTE ON THE TECHNIQUE OF SPHENOIDAL SINUS EXPLORATION FOR MENINGOCOCCAL AND OTHER INFECTIONS.

BY

P. WATSON-WILLIAMS, M.D. Lond.,

*Major, R.A.M.C.(T.);*
*Hon.Consultant for Diseases of Ear, Nose and Throat to the Military Hospitals, Southern Command.*

THE recent epidemics of cerebro spinal-meningitis lend importance to the question whether the nasal accessory sinuses, and more particularly the sphenoidal sinuses and posterior ethmoidal cells, are either occasionally or frequently the source from which the meninges become infected; in other words, provide the breeding-ground from which a local infection spreads so as to develop into cerebro-spinal meningitis. That the importance of exploration of the sphenoidal sinuses in conditions which might lead to or be caused by their infection is sometimes insufficiently recognised is perhaps due in part—

(*a*) To the erroneous assumption that the sinus is difficult of access for exploration, and

(*b*) To the belief that when infected the patient must suffer from some definite signs and symptoms that in most textbooks are described as characteristic.

One of the earliest recorded examples of meningococcal infection of the sphenoidal sinus proved in a living patient occurred in my own practice in the year 1900;[1] but the much more valuable and recent clinical research of Major Embleton and Major Peters[2] leaves no room for doubt that further attention to the sphenoidal sinus in cerebro-spinal meningitis may afford fruitful results in stamping out infection in carriers, and perhaps even in saving life. Embleton and Peters record post-mortem findings of patients dead from this disease where the sphenoidal sinuses were full of glairy pus, the mucosa so swollen and rugous as to render the ostia impervious to a probe, with osteitis of the bony walls of the sinuses and thick purulent lymph lying on the surface of the dura mater, corresponding with the sphenoidal sinus, the meningococcus being readily demonstrated both in the pus cells, in the sphenoidal sinus, and in the bone.

Lumbar puncture is regularly practised for the diagnosis of the disease, and frequently for its treatment; but surely if there is a priori reason to suspect that the sphenoidal

[1] *Diseases of the Upper Respiratory Tract,* Watson-Williams, 4th Edition, 1901, p. 366.

[2] Cerebro-spinal Fever and the Sphenoidal Sinus, *Lancet,* 1915, i. 1078.

sinuses are the source of the systemic infection in only a percentage of patients, one ought to explore also these cavities as a matter of diagnostic routine. And where these sinuses are shown to be definitely the seat of acute inflammatory swelling and exudation associated with those organisms, washing-out and drainage of the sinuses would be the natural course to adopt, unless experience showed that the procedure was unattended by favourable results. Exploration of the

FIG. 1.

*Section showing an unusually large and well-developed sphenoidal sinus which occupies almost the entire sphenoidal body and extends backwards so as to leave but a very thin plate of bone between the sinus and the foramen magnum. CO, optic canal; CI, prominence formed by the carotid canal on the outer wall of the sinus.* (*Sieur et Jacob.*)

sphenoidal sinus naturally requires care, knowledge of regional anatomy, and some technical experience, but the same may be said of lumbar puncture.

These recent and important observations recall the earlier work of other observers, notably Westenhœffer, who also regarded the sphenoidal sinuses as the most probable route, and Netter and Debre, who favoured the view that the meningococcus entered the subarachoid space either through the sheath of the olfactory nerve filaments as they passed through the cribriform plate or through other communications between the pia-arachnoid space and the root of the cells. It is not my intention to discuss the pathogenesis of cerebro-spinal meningitis, but to suggest the desirability of routine exploration of the sphenoidal sinuses in all cases in which the meningococcus has been found in cerebro-spinal fluid removed by lumbar puncture, and in those in which the organism has been found in the naso-pharynx and is associated with symptoms suggesting infection. It may not be out of place to emphasise the point that these sinuses may be infected by pyogenic organisms, and much mischief caused, although symptoms characteristic of sphenoidal sinusitis may be altogether absent, and even when no discharge can be seen in the nasal passages, at any rate by ordinary methods of anterior and posterior rhinoscopy. It must suffice to mention in illustration one group of this elusive latency, viz., the cases of canalicular optic neuritis with partial or even almost complete loss of vision, with negative nasal findings unless exploratory lavage and culture be employed. Now if, notwithstanding the fact that local nasal symptoms are insufficient to arrest attention, the nasal sinuses are sometimes the source of a toxic infection of the optic nerves, there is *a priori* very strong grounds for suspecting these sinuses as the breeding-ground for the meningococcus in cerebro-spinal meningitis, and this suspicion is confirmed by the references to recorded cases.

Discharges escaping from a sphenoidal sinus through the ostium sphenoidale must trickle down the external or anterior face of the anterior wall through the spheno-ethmoidal recess, and passing down between the nasal septum and posterior border of the middle turbinal on to the floor of the nose between the posterior end of the inferior turbinal body and septum nasi, thence over the back of the velum palati to drip and be swallowed or expectorated. Unless the source of the muco-pus is confused by the co-existence of purulent discharge from the frontal sinus or maxillary antrum, its origin in the posterior group of sinuses, viz., the posterior ethmoidal cells and sphenoidal sinus, is easy to conjecture. Whether one has to do either with ethmoidal

FIG. 2.

*Transverse section of the right nasal passage and orbital fossa. X, the right olfactory fissure between the septum nasi and the vertical plate of the middle turbinate bone; the sphenoidal sinus ostium lies at this level a little more posteriorly.* (*Shambaugh.*)

cell or sphenoidal sinus discharge, or, as is not seldom the case, a combination of both, can only be determined by direct inspection or by exclusion of sphenoidal sinus infection as the result of direct exploration.

When the mucus is abundantly mixed with polynuclears and the secretion is therefore obviously purulent or muco-purulent, the existence of abnormal discharge is usually obvious to the patient as well as to the medical attendant. But an infection of any of the nasal sinuses, and particularly of those of the posterior group (posterior ethmoidal cells and sphenoidal sinuses) is highly elusive, and much more difficult to determine when, owing to the relative paucity of polynuclears, the discharge is almost clear and pellucid as normal mucus. Yet such cases are prone to suffer the more from toxic absorption, and are more likely to spread by various routes. Without pushing the analogy too closely, one may compare the greater likelihood of a general septic infection from post-mortem wounds which do not form a local abscess. One must not assume that patients who have no obvious purulent discharge from the nasal passages, either anteriorly or post-nasally, cannot have infected nasal sinuses.

We now come to the essential purport of this communication, which is to describe the technique of exploring the sphenoidal sinuses with a view to proving by bacteriological methods the presence or absence of the diplococcus of Weichselbaum.

The ostium sphenoidale usually varies in size from two to three millimetres in the vertical diameter, and one to two millimetres in width, sometimes more, often less, and is situated nearer the upper than the lower border of the anterior wall of the sinus. But the anterior wall of the sinus is mainly formed by the very thin sphenoidal turbinal bone, and the bony orifice is usually much larger than the actual orifice in the mucous membrane, that is to say, the anterior wall of the sinus is to an appreciable extent mucous membrane only, and the remainder for the most part only very thin and bone about one thirty-second of an inch in thickness. The median third or half of the anterior wall corresponds with the olfactory fissure and spheno-ethmoidal recess, that is to say, it is free and faces the nasal fossa ; the other half or two thirds, according to the degree of development of the sinus, corresponds with the posterior wall of the posterior ethmoidal cells. The main point is that the inner portion of the anterior wall of the sphenoidal sinus is very thin, and is directly accessible through the nasal passages. It is usually impossible to inspect the ostium sphenoidale, as it lies a little to the outer side, so as to lie behind the inward projection of the middle turbinal body. Hence in passing a probe or small mop through the sphenoidal sinus ostium, one usually has to turn the point slightly outwards as soon as it impinges on the anterior face of the sinus wall. Even with such manœuvring, and by pressing and displacing the middle turbinate outwards, or else removing its anterior end, it is impossible to find the orifice and to enter the sinus in nearly fifty per cent. of cases without causing too much discomfort.

But experience has led me to the conclusion that if one desires a cultural investigation of the sinus contents, it is usually better to pass a cannula directly through the thin wall, and then suck out any secretions that may be lying in the sinus cavity, rather than try to find the natural ostium, and this for the following reasons :—

1. It is hardly possible to pass a mop or cannula into the sinus without previously or subsequently contaminating it while passing through the narrow olfactory fissure, in which case the culture yielded may be misleading.

2. If one uses a cannula and suction syringe, and the orifice of the sinus is small, the cannula so fills the ostium that neither air nor fluid can enter or escape and no contents can be withdrawn, as there is no " bunghole."

Usually local anæsthesia suffices, and of all local anæsthetics cocaine hydrochlorate is in my experience the best, firstly because it is the most efficient local anæsthetic, and, secondly, owing to its constricting the small vessels, and thus rendering the mucosa and the turbinal bodies anæmic. If adrenal extract is used at all, it should be a minute percentage added to the cocaine solution. The nasal passages should first be cleared by blowing the nose, or by spraying with some simple alkaline solution, *e.g.* common salt and bicarbonate of soda, so as to remove any secretions collected in the nasal passages. One fine spraying of 20 per cent. cocaine hydrochlorate aqueous solution should be made, directing it high up the nose into the olfactory fissure, and immediately making the patient lower his head, so that the solution may run out rather than backwards to the naso-pharynx. Then with a very fine absorbent wool mop wound on the end of a nasal probe, apply a little 20 to 30 per cent. cocaine solution to the anterior surface of the sphenoidal sinus, directing the cotton wool carrier high up in the olfactory fissure between the septum and the vertical plane of the middle turbinate. After two or three minutes' interval one may proceed to explore the sinus.

Exploration of the sinus may be by way of the natural ostium or by entry through the anterior wall. The patient may be seated in an ordinary chair with a head rest, or lying on the back. I prefer having the patient lying flat on his back, because it is easy to keep the head steady, and it has the effect of making the sphenoidal sinus opening uppermost, whereas if the head is erect the nozzle of the exploring cannula must dip down well below the level of the ostium before any of the sinus fluid contents can be sucked out, and not seldom this is impossible. It is quite as easy to make an inspection of the nasal passages and pass instruments with the patient lying on his back as in the more usual examination position; it is merely a question of habit and of having an adjustable lamp.

### A. ENTRY THROUGH THE NATURAL OSTIUM.

Take a sterilised short silver Eustachian catheter, No. 1 size, with the distal end for three-quarters of an inch bent downwards in a slight curve, the proximal end being of the usual size, and therefore fitting accurately the nozzle of the suction syringe. Pass the catheter under inspection by ordinary anterior rhinoscopy, using a sterile nasal speculum, and carry it upwards and backwards between the septum and anterior end of the middle turbinal, keeping close to the roof of the olfactory fissure till the nozzle impinges against the anterior sphenoidal sinus wall. Then turning the curved end slightly

FIG. 3.

*Section of the right nasal passages (outer wall), showing the method of passing a cannula or Eustachian catheter along the roof of the nasal passage till the distal end reaches the sphenoidal sinus ostium.* (*Sieur et Jacob.*)

outwards (instead of directly downwards as it was when introduced), feel for the sphenoidal sinus ostium by gentle tapping movements until by the sensation of the resistance disappearing the cannula is felt to have entered the sinus cavity. As soon as the cannula is in the sinus, one should try to make the downward curved nozzle in its further passage backwards get downwards towards the floor of the cavity. This manœuvre is *essential* if the patient's head is erect, since otherwise the end of the catheter does not lie sufficiently below the level of fluid in the sinus, which is determined by the position of the ostium : it is desirable with the patient on his back, as the deep part of the sinus cavity is below the level of the sella turcica.

In suggesting as above the line to try to follow for gaining access to the sinus ostium, I am aware that it cannot be done in all cases, because the anterior end of the middle turbinal body makes it impossible when it is so incurved as to approach very closely or actually impinge on the septum nasi. Yet even in such cases it is often easier to enter the probe high up above the inward curving of the turbinal than to do so from the lower level. In other cases, again, the high route is rendered impossible by anatomical conditions, and then it is usually easy to gain access to the ostium by introducing the probe or cannula below the obstruction. But in certain patients it is impossible to reach the goal either way, unless one can insert a long Killian speculum between the turbinal and the septum, and force the turbinal outwards, or, as a last resort, one may remove the obstruction afforded by the anterior end of the middle turbinate. The latter procedure should, I feel, be regarded as a *pis aller* in an exploration, first because it is rarely necessary, secondly because the nasal structures should not be removed unless absolutely necessary, and, thirdly, because if an infected sinus does exist, the less the vascular and lymphatic channels are laid open the better; and more particularly is this the case when one suspects the existence of a meningococcus infection which is anyhow prone to gain an entrance to the pia-arachnoidal space with dangerous or even fatal result.

### B. ENTRANCE THROUGH THE ANTERIOR WALL.

This is the route I have almost invariably followed, and in certainly at least a thousand sinuses the method of entry has proved easy and without any bad result. The blunt

PLATE I.

FIG. 4.

*Showing Watson-Williams's sphenoidal sinus cannula passed through the thin anterior wall of the sinus so as to lie well within the sinus cavity, the "patient" lying on the back. The arrow shows the position at the normal sinus ostium, S.O.*

trocar and cannula can almost invariably be insinuated between the middle turbinal body of the septum, and made to glide upwards and backwards so as to aim about one inch behind the centre of the back of the eyeball. On reaching the anterior face of the anterior sinus wall, the proximal end of the cannula should be raised to as to make it as nearly horizontal (assuming the head is erect) or as nearly vertical (assuming the patient is on his back) as possible, the distal end is gently pressed against the sinus wall, and moved slightly towards the upper or lower border of the wall, until it is felt to penetrate into the cavity, and then is gently inserted until it impinges against the posterior wall of the sinus. No force should be used, and the sensation of yielding to the slight pressure with the penetration into a cavity is so obvious that no doubt can be entertained of its being *in situ*. If it is considered possible that the cannula has passed below the sinus above the roof of the nasopharynx, one can settle the question by making a downward pressure of the canula, for if it be in the sinus, the thick bony floor of the sinus prevents such pressure displacing the distal end, while it is lying in the sinus.

Having previously drawn about 6 c.c. of sterile water into the sterilised suction syringe, the syringe nozzle is inserted carefully into the proximal end of the cannula, about 3 c.c. of the water is thrown into the sinus and at once slowly withdrawn. If the sinus contains a quantity of purulent discharge, it can be sucked into the syringe without using any water, provided the pus is not too thick; but as one cannot tell this beforehand, I always have some water in the syringe, and throw in 1 c.c. to 2 c.c., and then suck back, and then if necessary a little more, say 3 c.c., is thrown in and withdrawn. This makes it easier to withdraw any thick pus, and the pus, if present, can be seen as a turbid stream or sometimes as a coherent cord, like a piece of string or wool, running into the column of water in the syringe.

But if no pus can be seen in the fluid withdrawn from the sinus, but only a slight turbidity, it must not be assumed that the contents are healthy, the fluid should then be ejected from the syringe into a sterile bottle and submitted to bacteriological investigation. On the other hand, one must not assume that a little turbid mucus withdrawn from a sphenoidal sinus is conclusive evidence of its being infected or the seat of disease.

As a further precaution of practical value, the nozzle of the exploring cannula should not be in actual contact with the sinus wall during the suction process, as the mucous membrane may so hermetically seal the aperture that no suction of fluid is possible, and either nothing can be drawn back into the syringe, or only blood from the injured mucosa. To avoid this, having felt the nozzle impinge on the posterior wall of the sinus, one should withdraw it about one-eighth of an inch before making attempts to suck up the sinus contents.

FIG. 5.

*The author's exploring syringe with the sphenoidal sinus cannula attached. The two-way stop-cock shown in the diagram is now discarded, the nozzle of the syringe being attached directly to the cannula.*

It is quite possible in many cases to tell that the sphenoidal sinus mucous membrane is not healthy simply from the sensation of velvety thickening of the mucosa, which the impact of the cannula against the posterior sinus wall yields, instead of the feeling of coming against a bone covered with gold beater's skin, which is the normal sensation imparted. Moreover, the sphenoidal sinus lining mucosa may be more than velvety in the degree of thickening, it is fairly often distinctly œdematous, and occasionally polypoid; with such conditions it may be difficult or even impossible to withdraw fluid contents from the sinus, for although one can inject the water, as soon as one exerts any suction the œdematous mucous membrane tends to come against and seal the lumen of the cannula. The cavity of the sinus, too, may be so encroached on by an extremely swollen mucous membrane that there is little or no space for secretions to collect.

The fluid having been successfully collected, it should be examined (*a*) by staining films, (*b*) by culture.

The film examination allows cytological observations, and on staining the organisms present may be recognised, and any degree of phagocytosis noted.

If organisms are found in such quantity as to leave no doubt of the sinus under question being a seat of infection, the proper course is to open the sinuses by removing a considerable part of the anterior wall, so that free drainage is ensured and frequent lavage of the cavity becomes an easy matter. The method of opening and treating the sphenoidal sinus is outside the scope of this communication, and can only be undertaken with safety by those who have had special training in the operative treatment of the nasal passages.

Without attempting to discuss all the various symptoms and signs of a sphenoidal sinus infection, I would like to direct attention to the great value of the relatively new method of endo-rhinoscopy, more particularly in the diagnosis of sphenoidal sinus affections. The endo-rhinoscope is practically a periscope similar to a cystoscope, of a length and diameter suited to its use in the nasal passages. By ordinary anterior rhinoscopy it is very rarely possible to see this sphenoidal sinus ostium or the face of the anterior wall of the sinus in which it is situated, and one can never see the sphenoethmoidal fissure, and therefore one has hitherto depended on posterior rhinoscopy and the appearance of discharge coming down the choanæ narium above the posterior end of the middle turbinate. Now it is difficult to catch the discharge in that position, even when it comes down in sufficient quantity to be visible there; but although it is possible to see the pus in that position, one has still to surmise that what is seen must have come from the posterior group of cells, valuable as that may be as far as it goes. But with the endo-rhinoscope one can get a direct view of all the region, and look at the Eustachian tubes and above them into the sphenoethmoidal recess, observing strings or small blobs and fibres of muco-pus that by any other means would altogether escape notice. One great advantage of endo-rhinoscopy lies in the ease with which one can make direct inspection in a patient who is too ill to be examined by posterior rhinoscopy, or one has only to pass the instrument along the floor of the nasal passages until the posterior border of the vomer comes into view, and then rotate it gradually so as to bring the various parts under inspection. This can be done as the patient lies on his back in bed as easily as in the sitting posture, and in an unconscious or semi-conscious patient in whom any other method of inspection is either impossible or highly unsatisfactory. But owing to the nature of the infection it is essential to wear an efficient nose and mouth mask in cases suspected of cerebo-spinal meningitis.

FIG. 6.

*Transverse section through the sphenoidal sinuses. The dividing septum is displaced to the left, the right sinus bein -large, and it would be possible to enter this sphenoidal sinus through either nasal passage N.opt, optic nerve ; Pr, prolongation of the sinus into the anterior clinoid process and lesser wing ; III, IV, VI, third, fourth and sixth nerves ; $V^1$ and $V^2$, the first and second branches of the Vth nerve.* (*Sieur et Jacob.*)

FIG. 7.

*Transverse section of the nasal passages through the sphenoidal sinuses. In this preparation the right sphenoidal sinus (S.sp.d.) and the left (S.sp.g.) are irregularly developed, and especially on the left side the posterior ethmoidal cells are extending backwards and encroaching on the space usually occupied by the sphenoidal sinus* (*Sieur et Jacob.*)

A further point that it may be worthy to note here is the fact that a headache due to sphenoidal sinusitis is rarely located at the seat of origin. Headache is frequently absent, and when present is usually located at the back of the eyeball, and not seldom is either supra-orbital, suggestive of a frontal sinus affection, or in the ear of the corresponding side where it may be so severe that the patient is convinced that he has an abscess there. The difficulty in determining the existence or otherwise of infective sphenoidal sinusitis in its early stage is often very considerable, and may remain for some time in doubt, unless one resorts to the clinching test of exploration and culture; and more particularly is it desirable that in cerebro-spinal meningitis the suspicion should be cleared up before the progress of the disease has rendered such information useless to the patient.

I find that during the first half of the year I have explored fifty-one sphenoidal sinuses in my private practice, besides a considerable number at my hospital clinic, but of these fifty-one sinuses twenty were bilateral explorations. Operation on the sphenoidal sinuses in the majority of cases so explored was not performed as the results of exploration, were negative or too indefinite to justify it. Many of the explorations were carried out under general anæsthesia for operations on other sinuses that were known to be diseased, or on the septum, and a considerable proportion were done under simple cocaine anæsthesia. The latter were sometimes described by the patient as distinctly painful, though in no case did they complain much or show signs of pain at the time, nor did pain continue after the procedure was completed, the worst feature being in a few patients a slight headache lasting an hour or two.

MEASUREMENTS.

The distance between the point of entrance to the nasal passages and the anterior and posterior walls of sphenoidal sinus vary considerably, and more especially so as regards the posterior wall. One's approach to the sphenoidal sinus is along an oblique line, and the face of the anterior sphenoidal sinus wall is also oblique, hence the measurement in any given case depends to some extent whether one strikes the sinus in its upper or lower part. This variation is greater when one comes to the posterior wall, because a straight cannula passing through the natural ostium or through the upper portion of the anterior wall usually comes up against the wall of the sella turcica, that is the shallow part of the sinus, whereas in passing through the lower half of the anterior wall one enters the deeper part of the sinus. One can usually reach the deeper part of the sinus even with a cannula entering through the upper part of the anterior sinus wall, (*a*) either by using a cannula with a downward curve or (*b*) by raising the proximal end of the straight cannula till the entering instrument is as nearly horizontal as the nose permits, taking care to keep the distal end steady and unshifting from the point where it rests against the anterior sinus wall. The latter manœuvre anyhow has a double advantage; firstly, it carries one into the deep part of the sinus, and secondly, after entering the sinus one comes up against the thicker part of the posterior sinus wall instead of the relatively thin anterior wall of the sella turcica or perhaps the roof. Anyhow, it is wise not only to keep the cannula as horizontal as one can, but also to keep along the side of the septum and not direct the blunt end of the exploring cannula outwards, where it might come against the outer wall, and where careless manipulation and any unnecessary force might injure the cavernous sinus wall or even the optic nerve.

I usually make my measurements from the tip of the patient's nose to the anterior wall and to the posterior wall of the sinus, the difference giving one the depth of the sinus.

I have made a large number of measurements from the anterior surface of the upper tip at its junction with the lower margin of the naris, but this is less convenient.

Taking a few cases at random, I find the measurements are :—

| *Adults.* | *Nose tip to anterior wall.* | *Nose tip to posterior wall.* |
|---|---|---|
| Male .. .. .. .. | — | R. $3\frac{3}{4}$, L. $3\frac{1}{2}$. |
| Male .. .. .. .. | — | R. $3\frac{1}{4}$. |
| Male .. .. .. .. | — | R. $3\frac{7}{8}$, L. $3\frac{5}{8}$. |
| Male .. .. .. .. | R. $3\frac{1}{3}$, L. $3\frac{1}{3}$. | R. 4, L. $4\frac{1}{8}$. |
| Female .. .. .. | R. $3\frac{1}{8}$, L. $3\frac{1}{8}$. | R. $3\frac{7}{8}$, L. $3\frac{7}{8}$, |
| Female .. .. .. | — | R. $3\frac{7}{8}$, L. 4. |
| Female .. .. .. | — | L. $3\frac{1}{8}$. |
| Female .. .. .. | — | R. $3\frac{1}{2}$, L. $3\frac{1}{2}$. |
| Female .. .. .. | — | R. $3\frac{1}{2}$, L. $3\frac{2}{5}$. |
| Female .. .. .. | — | R. $3\frac{7}{8}$, L. 4. |
| Female .. .. .. | $2\frac{7}{8}$. | $3\frac{1}{8}$. |

In a girl aged $14\frac{1}{2}$ years the distance was $2\frac{1}{2}$ inches to the anterior wall R. and L., and to the posterior wall R. $3\frac{1}{3}$, L. $3\frac{1}{2}$.

Others give measurements as follows :—

Rosenberg, [1] from the anterior naris to the anterior sinus wall .. .. .. .. .. 6 to 8 cm.

Laurent, [2] from the anterior nasal spine to the anterior sinus wall .. .. .. .. .. $6\frac{1}{2}$ cm.

Sieur et Jacob, [3] from the anterior nasal spine to the anterior nasal wall .. .. .. .. 7 to 8 cm.

I prefer to rely on my own above-mentioned observations, as they are without exception those obtained clinically. But these measurements must not be taken as giving the real depth of the sinuses between the anterior and posterior walls,

[1] Soc. de Laryng. de Berlin, 1894, cited by Sieur et Jacob.

[2] Reunion anuuelle des Otolaryngologistes belges, June, 1894, cited by Sieur et Jacob.

[3] *Fosses Nasales*, p. 346.

such as one could get from the same subjects if they were measured in sagittal section in anatomical specimens, the "errors" being due to the causes previously described.

---

### EXPERIENCES OF AN EAR, NOSE AND THROAT SPECIALIST AT ONE OF THE BASES.

## Figures and Tables

**Fig. 1. f1:**
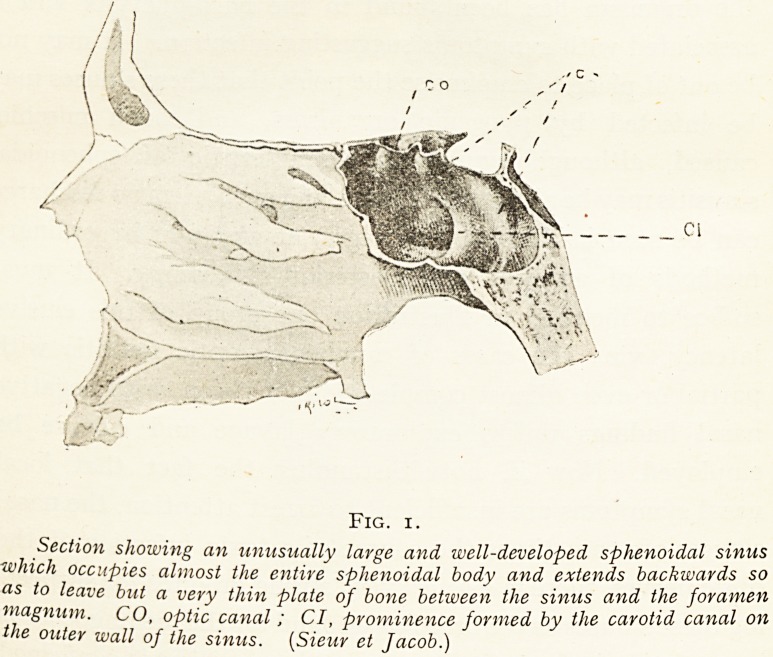


**Fig. 2. f2:**
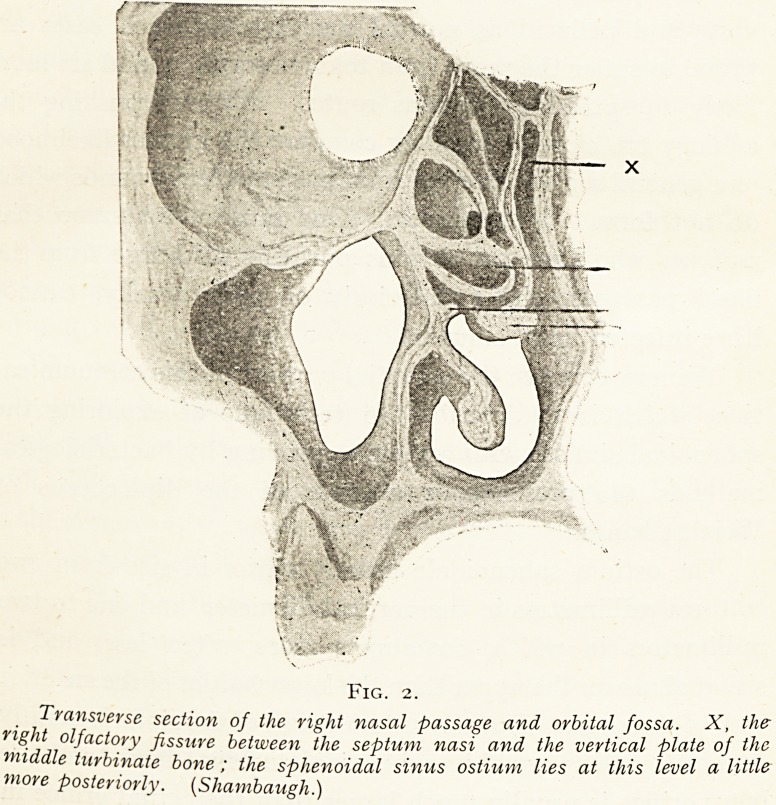


**Fig. 3. f3:**
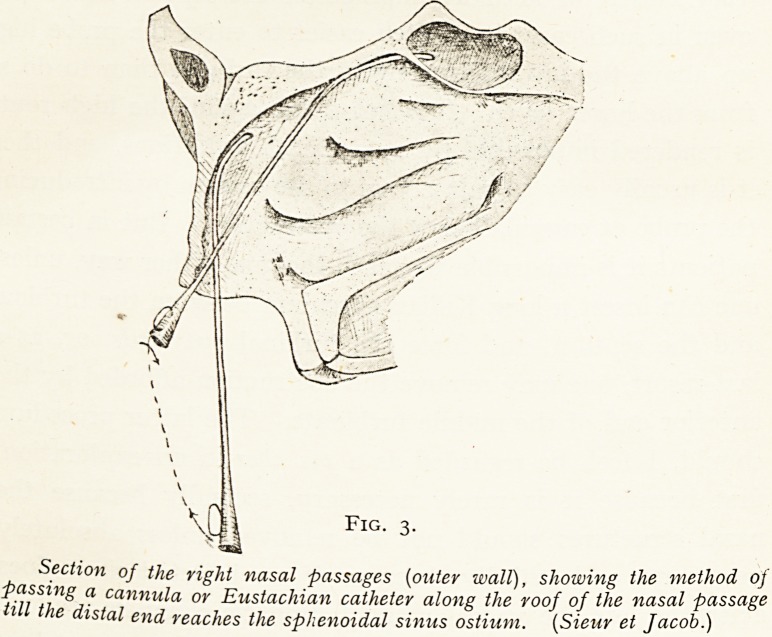


**Fig. 4. f4:**
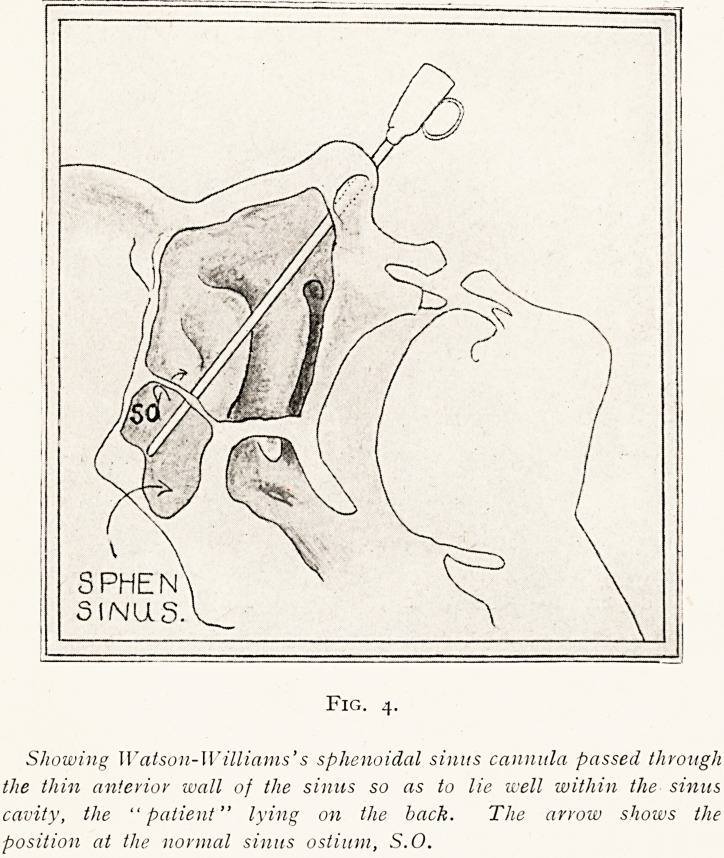


**Fig. 5. f5:**
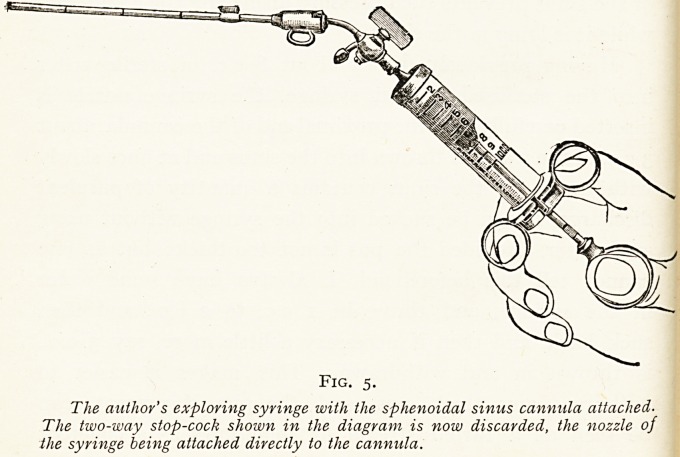


**Fig. 6. f6:**
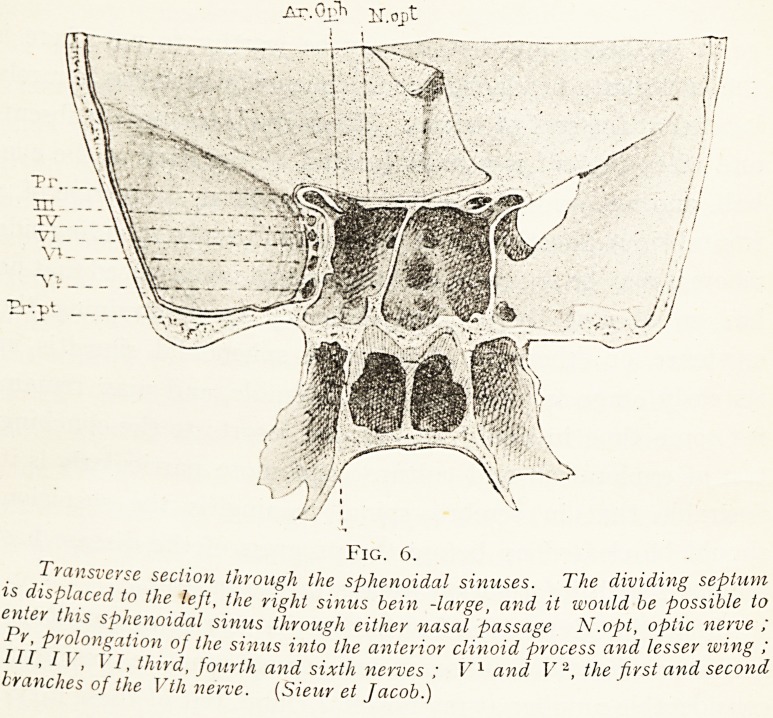


**Fig. 7. f7:**